# Exercise-Induced Dehydration Limits the Effectiveness of Post-Exercise Whey Protein Supplementation in Healthy Young Men

**DOI:** 10.3390/nu18193267

**Published:** 2026-10-04

**Authors:** Zekun Xin, Mengtong Gao, Xinjie Wu, Min See, Peng Cheng, Yi Yan

**Affiliations:** 1 Department of Sport Biochemistry, School of Sport Science, Beijing Sport University, Beijing 100084, China; xinzekun121@bsu.edu.cn (Z.X.); 2026210336@bsu.edu.cn (M.G.); 2024012764@bsu.edu.cn (X.W.); seemin3009@gmail.com (M.S.); 2 China Institute of Sport and Health Science, Beijing Sport University, Beijing 100084, China; chengpeng529@126.com; 3 Key Laboratory of Sports Stress and Adaptation, General Administration of Sport, Beijing Sport University, Beijing 100084, China

**Keywords:** exercise-induced dehydration, fluid restriction, whey protein, serum amino acids, branched-chain amino acids

## Abstract

Background/Objectives: Post-exercise protein ingestion is widely used to promote recovery from exercise, but whether exercise-induced dehydration limits the subsequent protein supplementation response remains unclear. This study examined the effects of different hydration strategies during exercise on post-exercise whey protein supplementation. Methods: Thirteen healthy young men completed three partially crossover trials designed to produce approximately 2% (DEH: a dehydration trial without fluid replacement), 1% (EUH_50%: a 50% fluid-replacement trial), and 0% (EUH: a euhydration trial with 100% fluid replacement) body mass loss. Within 5 min after each exercise, participants ingested 45 g of whey protein dissolved in 450 mL of water. Serum amino acid concentrations were measured before exercise, immediately after exercise, and at 60 and 120 min after ingestion. Results: The protocol produced distinct exercise hydration status, with body mass loss of 2.20 ± 0.19% (DEH), 1.18 ± 0.30% (EUH_50%), and 0.27 ± 0.35% (EUH), respectively. Post-exercise whey protein ingestion increased serum amino acid concentrations in all trials. However, at 60 min after ingestion, the DEH trial showed lower total amino acid, branched-chain amino acid, leucine, isoleucine, and glutamine concentrations than both EUH_50% and EUH (all *p* < 0.05). The 0–120 min percentage incremental area under the curve for total amino acids, branched-chain amino acids, isoleucine, and glutamine was also lower in DEH. Gastrointestinal symptoms remained low across all conditions, with only 1.0% of individual item scores exceeding 4. Conclusions: These findings indicate that exercise-induced hypohydration of approximately 2.2% body mass attenuates the early circulating amino acid response to post-exercise whey protein ingestion, suggesting that inadequate fluid intake during exercise may compromise the efficacy of whey protein supplementation for post-exercise recovery.

## 1. Introduction

Whey protein is widely utilized for post-exercise skeletal muscle remodeling [1,2,3,4] and training recovery [2,3,4,5], owing to its complete profile of essential amino acids, its exceptionally high concentration of branched-chain amino acids (BCAAs), and its rapid digestibility and absorbability. In a survey of 527 elite athletes, protein products were the most frequently reported supplement category and were used by 41% of supplement users [6,7].

Proper hydration is an essential prerequisite for the efficient digestion, absorption, and transport of nutrients. Hypohydration not only imposes additional cardiovascular and thermoregulatory stress [8,9,10], but also slows gastric emptying and increases gastrointestinal discomfort [11]. However, previous studies on whey protein supplementation have primarily focused on protein source, dose, timing, and distribution patterns [4,12,13,14], or have examined whether protein-containing beverages improve post-exercise fluid retention or rehydration [15,16,17]; yet none have considered the influence of hydration status on the response to whey protein supplementation. Athletes typically lose approximately 1.1 ± 0.07% of their body mass (BM) during training, with the incidence of exercise-induced dehydration ranging from 30% to 97% [18,19]. To investigate the effect of different degrees of dehydration on the efficiency of whey protein supplementation, the present study compared serum amino acid responses after consuming the same amount of whey protein following exercise-induced BM losses of approximately 2%, 1%, and 0% (no loss). The aim was to provide experimental evidence for improving the efficacy and rational use of whey protein supplementation. We hypothesized that the greater the degree of exercise-induced hypohydration, the weaker the early-phase serum total amino acid and BCAA responses would be.

## 2. Materials and Methods

### 2.1. Subjects

Thirteen healthy young male participants were recruited for this study (age: 22.7 ± 3.3 years, BM: 77.5 ± 9.5 kg, BM index: 23.7 ± 1.9 kg·m^−2^). An a priori power analysis was performed using G*Power version 3.1.9.7 (University of Kiel, Kiel, Germany) [20]. The analysis indicated that 11 participants were required to achieve 85% power with an effect size of 0.25 and α = 0.05.

Participants were eligible if they were nonsmokers, were not professionally trained, exercised habitually no more than three times per week, had no history of lactose intolerance or milk-protein allergy, and had no known neuromuscular, metabolic, gastrointestinal, or cardiovascular disease. Exclusion criteria were use of nutritional supplements within 1 month before enrollment, participation in another nutrition or exercise intervention during the study period, use of medications known to affect protein or amino acid metabolism, or inability to comply with the experimental procedures.

The study protocol was approved by the Ethics Committee of Beijing Sport University (approval No. 2025294H). All participants were informed of the experimental procedures and potential risks and provided written informed consent before participation. All procedures were conducted in accordance with the Declaration of Helsinki [21].

### 2.2. Study Design

This study used a self-controlled, partially randomized crossover design, with reporting guided by principles for crossover trials [22]. Each participant completed three experimental trials: a dehydration trial without fluid replacement (DEH), a 50% fluid-replacement trial (EUH_50%), and a euhydration trial with 100% fluid replacement (EUH). Trials were separated by at least 7 days. The DEH trial was completed first to determine the exercise duration required to induce approximately 2% BM loss during standardized treadmill running and to establish each participant’s individual absolute BM loss. Thereafter, participants completed the EUH_50% and EUH trials in randomized order. In the EUH_50% and EUH trials, participants followed the exercise protocol established in DEH while ingesting water during exercise equivalent to 50% and 100%, respectively, of the absolute BM loss recorded in DEH.

### 2.3. Pre-Trial Standardization

Participants refrained from strenuous exercise for 48 h before each trial and maintained their habitual sleep schedule. Dietary intake was recorded for 2 days before the first trial using the Bohee Health mobile application (Shanghai Bohee Health Technology Co., Ltd., Shanghai, China) and replicated before subsequent trials. On the day preceding each experimental trial, each participant was provided with 2 L of water and instructed to consume the entire volume in several portions throughout the day to standardize pre-trial fluid intake [23]. Approximately 2 h before exercise, they consumed a standardized breakfast providing ~7 kcal/kg BM (67.3% carbohydrate, 7.6% protein, and 24.8% fat) and 500 mL water [13]. On arrival, urine specific gravity (USG) was measured using an AutoFlex R817 refractometer (Rudolph Research Analytical, Hackettstown, NJ, USA). Trials proceeded only when USG was <1.020; otherwise, they were rescheduled [24]. Participants emptied their bladder and were weighed nude and dry to determine pre-exercise BM (BMpre). A baseline venous blood sample was then collected. All trials were conducted between 07:00 and 12:00. Laboratory temperature and relative humidity were approximately 25 °C and 61%, respectively.

### 2.4. Experimental Protocol

**Figure 1:**
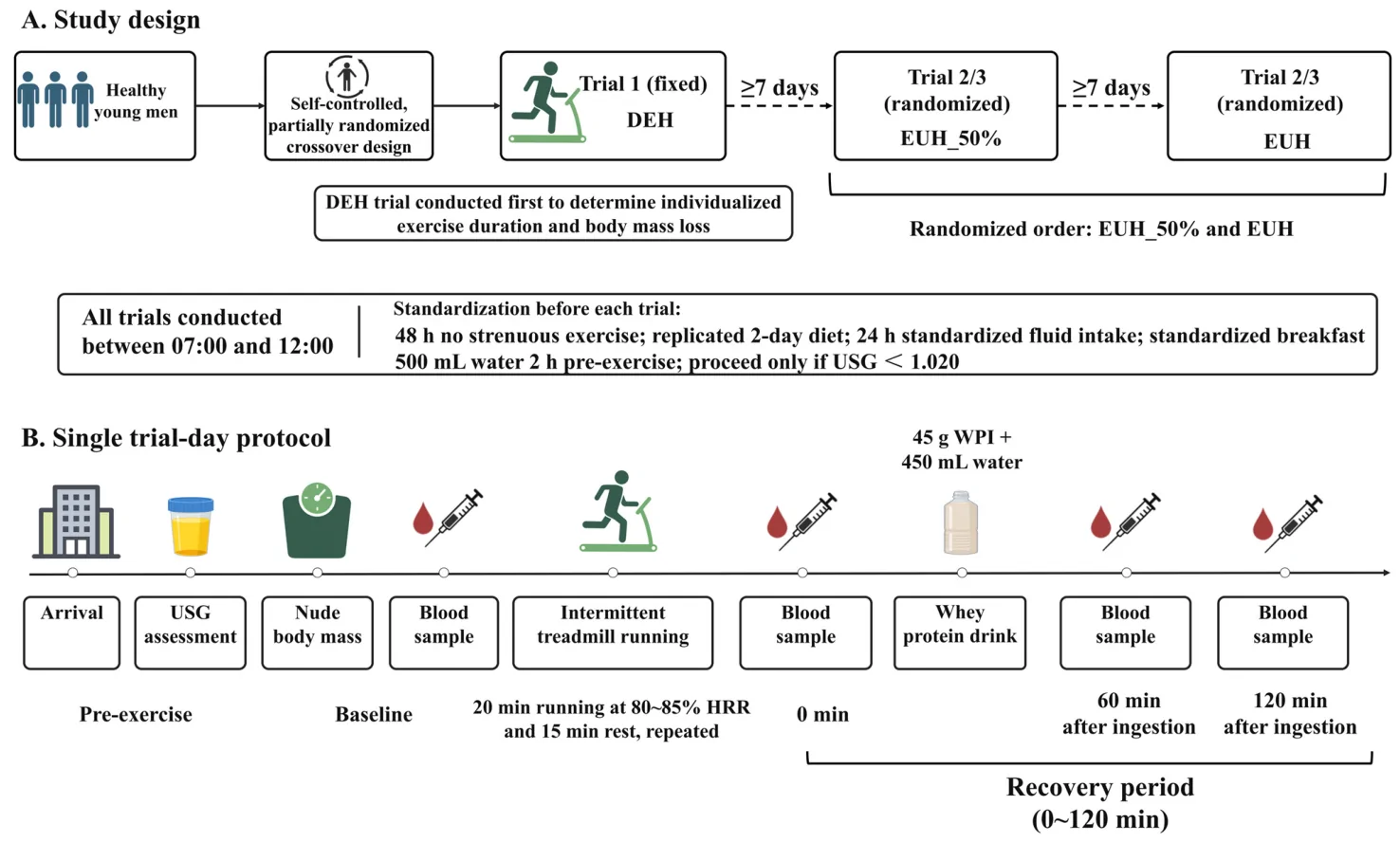
Study design and experimental timeline. (**A**) Within-participant, partially randomized crossover design. DEH was completed first to establish each participant’s individualized exercise duration and absolute BM loss; EUH_50% and EUH were subsequently completed in randomized order, with at least 7 days between trials. (**B**) Trial-day protocol. Pre-exercise hydration was confirmed by USG < 1.020. Participants then completed the individualized intermittent treadmill protocol. A venous blood sample was obtained immediately after exercise (0 min), followed within 5 min by ingestion of 45 g whey protein isolate dissolved in 450 mL water; additional blood samples were collected at 60 and 120 min after ingestion. No additional food or fluid was permitted during the 120-min recovery period. DEH, dehydration trial; EUH_50%, 50% fluid replacement trial; EUH, 100% fluid replacement trial; USG, urine specific gravity; HRR, heart rate reserve; WPI, whey protein isolate.

In the DEH trial, participants performed repeated 20-min bouts of treadmill running, separated by 15-min rest periods for nude BM reassessment; these 20-min running bouts were repeated without fluid intake until BM loss reached ∼2% BM_pre_ [25] (Figure 1). Upon exercise completion, participants were weighed nude and dry again (BM_postexer_). Running intensity was prescribed at 80~85% of heart rate reserve using the Karvonen method [26], and heart rate was monitored continuously with a Polar H10 sensor (Polar Electro Oy, Kempele, Finland). In EUH_50% and EUH, participants followed the same individualized exercise protocol established in DEH while ingesting water equivalent to 50% (725~1100 mL) and 100% (1450~2200 mL), respectively, of the absolute BM loss recorded in the DEH trial. Participants ingested the prescribed water volume in equal aliquots at 5-min intervals from 5 min before exercise until the end of the final bout, including during interbout rest periods [8,9,10,27,28,29,30,31,32].

Immediately after the exercise, 5 mL venous blood samples were collected from the subjects, and within 5 min, they were given 450 mL of a solution containing 45 g of isolated whey protein (Lacprodan DI-BLG 100 Neutral, Arla Foods Ingredients, Aarhus, Denmark) [33]. Additional venous blood samples were collected at 60 and 120 min later. During the 120-min recovery period, no additional food or fluid was allowed. Gastrointestinal symptoms were assessed immediately after ingestion and at 60 and 120 min [34].

### 2.5. Sweat Collection and Electrolyte Analysis

Investigators collected local sweat samples from each participant’s left forearm during treadmill exercise using an absorbent-patch method based on established procedures [27,35,36]. Before each application, the skin was cleaned with alcohol, rinsed with deionized water, and dried with sterile gauze. A 5 × 7 cm patch (absorbent area, 2.5 × 4 cm) was applied at the start of each 20-min running bout and removed during the subsequent rest period. Patches were weighed before and after application, and local sweat rate was calculated from sweat mass, absorbent area, and application time. Sweat was recovered by centrifugation, and sodium, potassium, and chloride concentrations were measured with an EasyLyte analyzer (Medica Corporation, Bedford, MA, USA).

### 2.6. Perceptual Responses and Gastrointestinal Symptoms

Ratings of perceived exertion (RPE) were recorded using the Borg 6–20 scale [37], and thirst sensation was recorded using a 1–9 numerical rating scale [38]. RPE and thirst sensation were assessed before exercise, immediately after exercise, and 60 and 120 min after whey protein ingestion. During exercise, both variables were also recorded every 5 min within each 20-min running bout.

Gastrointestinal symptoms were assessed using an adapted exercise gastrointestinal symptom questionnaire [25,34,39]. Each item was scored from 0 (no problem) to 9 (the worst it has ever been). The questionnaire included upper gastrointestinal symptoms (reflux, heartburn, vomiting, nausea, thick saliva, and belching), lower gastrointestinal symptoms (intestinal cramps, abdominal pain, bloating/flatulence, and urge to defecate), and systemic symptoms (muscle cramping, headache, urge to urinate, and dizziness). Scores > 4 were classified as serious symptoms [39].

### 2.7. Serum Amino Acid Analysis

Blood samples were collected into serum tubes, allowed to clot at room temperature, centrifuged at 4 °C, aliquoted, and stored at −80 °C until analysis. Serum amino acid concentrations were determined by liquid chromatography–tandem mass spectrometry (LC–MS/MS), following sample preparation procedures commonly used for the analysis of free amino acids in human serum [40,41]. For sample preparation, serum samples were diluted 20-fold with deionized water. Proteins were precipitated by adding methanol/acetonitrile (2:1, *v*/*v*) at a serum-to-precipitation-solvent ratio of 1:4. After thorough vortex mixing, the samples were incubated at −40 °C for 2 h and subsequently centrifuged at 14,000× *g* for 15 min at 4 °C. The supernatant was collected and passed through a 0.22-μm polytetrafluoroethylene (PTFE) syringe filter, and 100 μL of the filtrate was transferred to an autosampler vial for LC–MS/MS analysis. LC–MS/MS analysis was performed using an LC-20A liquid chromatography system coupled to an LCMS-8050 triple-quadrupole mass spectrometer (Shimadzu, Kyoto, Japan). Chromatographic separation was achieved on an InertSustain AQ-C18 column (100 × 2.1 mm I.D., 1.9 μm; GL Sciences Inc., Tokyo, Japan). Amino acids were quantified by external calibration using calibration curves prepared from a 21-amino-acid mixed standard (Shanghai Yuanye Bio-Technology Co., Ltd., Shanghai, China).

### 2.8. Statistical Analysis

Data are presented as mean ± SD. Single-value outcomes were compared among conditions using one-way repeated-measures analysis of variance (ANOVA). Time-series outcomes were analyzed using two-way repeated-measures ANOVA with condition and time as within-participant factors. When sphericity was violated, Greenhouse–Geisser-adjusted results were reported. Significant main or interaction effects were followed by Bonferroni-adjusted pairwise comparisons. Gastrointestinal symptom data were summarized as medians and observed maxima and were evaluated across condition and time with participant included as a repeated factor. Percentage incremental area under the curve (%iAUC) was calculated by the trapezoidal method from the percentage change at 60 and 120 min relative to the immediate post-exercise value [42,43,44]. Statistical analyses were performed using IBM SPSS Statistics version 27.0 (IBM Corp., Armonk, NY, USA).

## 3. Results

### 3.1. Hydration Manipulation

The intervention produced three distinct exercise hydration states (Table 1). Absolute BM loss differed among conditions (DEH, 1.72 ± 0.20 kg; EUH_50%, 0.93 ± 0.26 kg; EUH, 0.22 ± 0.28 kg; F = 167.86, *p* < 0.001), as did relative BM loss (DEH, 2.20 ± 0.19%; EUH_50%, 1.18 ± 0.30%; EUH, 0.27 ± 0.35%; F = 149.28, *p* < 0.001); all pairwise comparisons were significant (all *p* < 0.001). Pre-exercise USG was similar across DEH, EUH_50%, and EUH (1.014 ± 0.005, 1.014 ± 0.004, and 1.013 ± 0.005, respectively). Post-exercise USG values were 1.025 ± 0.003, 1.015 ± 0.007, and 1.009 ± 0.008, respectively.

Mean exercise heart rate did not differ among conditions (F = 2.71, *p* = 0.080). Local sweat rate and sweat sodium, potassium, and chloride concentrations were also similar across conditions (all *p* > 0.05). Thirst differentiated the hydration conditions. Mean exercise thirst was higher in DEH than in EUH_50% (*p* = 0.009) and EUH (*p* = 0.014), with no difference between EUH_50% and EUH (*p* = 0.261). Thirst did not differ among conditions before exercise. Immediately after exercise, thirst was higher in DEH (6.15 ± 1.72) than in EUH_50% (4.46 ± 1.56; *p* = 0.050) and EUH (3.62 ± 1.85; *p* = 0.002), and this difference persisted at 60 min (5.92 ± 2.10 vs. 3.00 ± 1.47 and 2.92 ± 1.61, respectively; both *p* < 0.001) and 120 min (6.62 ± 2.02 vs. 2.69 ± 1.70 and 3.00 ± 1.73, respectively; both *p* < 0.001). EUH_50% and EUH did not differ at any recovery time point.

### 3.2. Ratings of Perceived Exertion (RPE) and Gastrointestinal Symptoms

Mean RPE during exercise was similar among DEH, EUH_50%, and EUH (14.6 ± 1.9, 14.0 ± 1.5, and 14.1 ± 1.7, respectively; F = 0.44, *p* = 0.648). RPE measured before exercise and during postexercise recovery showed significant effects of time (F = 213.82, *p* < 0.001) and condition (F = 8.72, *p* = 0.001), but no condition × time interaction (F = 0.51, *p* = 0.758). RPE did not differ among conditions before exercise. Compared with EUH, RPE was higher in DEH immediately after exercise (16.46 ± 1.98 vs. 14.08 ± 2.43; *p* = 0.024), at 60 min (8.54 ± 2.33 vs. 6.77 ± 0.73; *p* = 0.022), and at 120 min (7.46 ± 1.81 vs. 6.08 ± 0.28; *p* = 0.011). At 120 min, RPE was also higher in DEH than in EUH_50% (6.31 ± 0.75; *p* = 0.043); no other between-condition differences were observed.

GI symptom scores after whey protein ingestion were low across all trials (Table 2). The total GI symptom score changed over time (F = 10.59, *p* < 0.001), but there was no condition effect (*p* = 0.575) or condition × time interaction (*p* = 0.736). Individual item scores > 4 were uncommon (17 of 1638 item-level ratings, 1.0%), indicating no condition-specific GI intolerance to the post-exercise whey protein beverage.

### 3.3. Serum Amino Acid Responses After Whey Protein Ingestion

**Figure 2:**
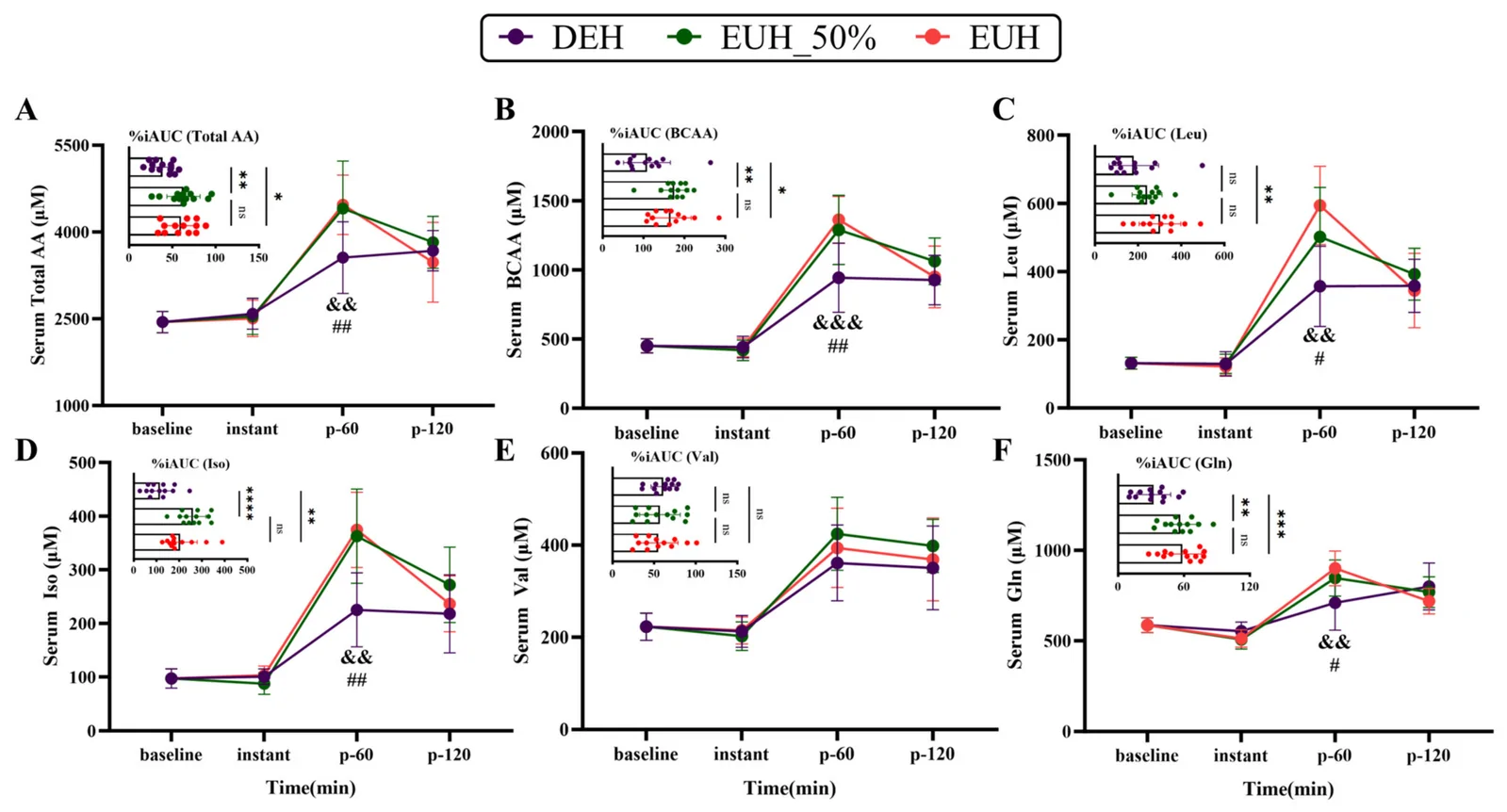
Changes in serum amino acid concentrations and %iAUC after whey protein ingestion in different states of exercise-induced dehydration. Panels show total amino acids (**A**), BCAAs (**B**), leucine (**C**), isoleucine (**D**), valine (**E**), and glutamine (**F**). Time points are baseline, immediately post-exercise before whey protein ingestion (instant), 60 min post-ingestion, and 120 min post-ingestion. Data are presented as means ± SD. %iAUC was calculated by the trapezoidal method using the percentage change at 60 and 120 min relative to the 0-min value, with the 0-min value constrained to 0. DEH, dehydration trial; EUH_50%, 50% fluid replacement trial; EUH, 100% fluid replacement trial. At 60 min, &&, and &&& indicate *p* < 0.01, and *p* < 0.001, respectively, for comparisons between EUH and DEH, and # and ## indicate *p* < 0.05 and *p* < 0.01, respectively, for comparisons between EUH_50% and DEH. In the %iAUC insets, horizontal brackets indicate pairwise comparisons; ns indicates no significant difference; *, **, *** and **** indicate *p* < 0.05, *p* < 0.01, *p* < 0.001 and *p* < 0.0001, respectively.

Serum amino acid concentration–time responses and %iAUC are presented in Figure 2. Serum concentrations of total amino acids, BCAAs, leucine, isoleucine, valine, and glutamine increased over time following whey protein ingestion (all time effects, *p* < 0.001). Significant condition × time interactions were observed for total amino acids, BCAAs, leucine, isoleucine, and glutamine (all *p* < 0.001), but not for valine (*p* = 0.270). Between-condition differences were primarily evident at 60 min after ingestion. At this time point, serum total amino acid, BCAA, leucine, isoleucine, and glutamine concentrations were lower in DEH than in EUH_50% and EUH, whereas no differences were observed between EUH_50% and EUH. The %iAUC also differed among conditions for total amino acids (F = 7.85, *p* = 0.001), BCAAs (F = 7.08, *p* = 0.003), leucine (F = 5.25, *p* = 0.010), isoleucine (F = 16.50, *p* < 0.001), and glutamine (F = 10.73, *p* < 0.001), but not for valine (F = 0.24, *p* = 0.787). Pairwise comparisons showed that %iAUC values for total amino acids, BCAAs, isoleucine, and glutamine were lower in DEH than in both EUH_50% and EUH. Leucine %iAUC was lower in DEH than in EUH, but did not differ between DEH and EUH_50%. None of the %iAUC outcomes differed between EUH_50% and EUH.

## 4. Discussion

The primary finding was that exercise-induced hypohydration of approximately 2.2% of BM significantly attenuated the early post-prandial serum amino acid response following whey protein ingestion, as reflected by lower concentrations at 60 min and reduced 0–120 min %iAUC for total amino acids, BCAAs, isoleucine, and glutamine. Notably, this impairment was already evident at a dehydration level of 2.2% BM loss—well within the range commonly observed during routine training (approximately 1.1 ± 0.07%) and competitive endurance events [18]. Furthermore, increasing fluid replacement from 50% to 100% of exercise-induced fluid losses did not yield additional benefits in amino acid availability, suggesting a potential threshold effect whereby avoiding complete fluid restriction, rather than achieving full fluid replacement, may be sufficient to preserve the early post-exercise amino acid response. These findings provide novel evidence that hydration status during exercise is a previously overlooked modifiable factor influencing the efficacy of post-exercise whey protein supplementation.

### 4.1. Hydration Manipulation and Perceptual Responses

The fluid replacement strategies employed during exercise successfully produced three distinct post-exercise hydration states, with BM losses of 2.20 ± 0.19% in DEH, 1.18 ± 0.30% in EUH_50%, and 0.27 ± 0.35% in EUH. Mean heart rate and RPE during exercise did not differ significantly across conditions in a moderate environment relevant to routine training (approximately 25 °C and 61% relative humidity), which is consistent with the findings of Wall et al., who reported little separation in cardiovascular or perceptual responses between trials with 0%, 2%, and 3% BM loss under conditions of substantial convective cooling [45]. The meta-analysis by Deshayes et al. also found that the mean increase in RPE per 1% BM loss was small and highly variable across exercise settings [46]. The repeated 20-min running bouts with 15-min rest intervals and the moderate laboratory environment (approximately 25 °C) in the present study may have attenuated the progressive accumulation of cardiovascular and thermoregulatory strain typically observed during continuous exercise in the heat [47,48], thereby limiting the separation of RPE and heart rate despite the targeted fluid deficit.

In contrast to RPE and heart rate, thirst sensation clearly differentiated the hydration conditions. DEH demonstrated consistently higher thirst than both EUH_50% and EUH during exercise and throughout the 120-min recovery period. This observation aligns with the well-established role of thirst as a sensitive, albeit imperfect, indicator of body water deficit [38]. The persistent elevation of thirst in DEH during recovery, despite whey protein beverage ingestion, suggests that the 450 mL of fluid provided with the protein supplement was insufficient to correct the 1.72 L fluid deficit induced in DEH. This finding has practical implications: athletes who complete exercise in a significantly dehydrated state should be aware that post-exercise protein beverages alone may not adequately address fluid restoration needs, and separate rehydration strategies should be considered.

### 4.2. Gastrointestinal Tolerance and Its Interpretation

Gastrointestinal symptoms remained low across all conditions, with only 1.0% of individual item scores exceeding 4. This observation is noteworthy for two reasons. First, it confirms that the 45 g dose of whey protein isolate dissolved in 450 mL water was well tolerated even under dehydrated conditions, supporting the feasibility of this supplementation strategy in real-world athletic settings where mild-to-moderate dehydration is common. Second, the absence of condition-specific GI symptom differences despite clear separation in amino acid responses suggests that the reduced amino acid availability in DEH was not mediated by overt GI distress—a point that parallels the findings of Costa et al., who reported greater intestinal injury and carbohydrate malabsorption with 3.1% dehydration in the absence of increased GI symptom severity [49]. Collectively, these findings indicate that subjective GI symptom questionnaires alone are insufficient to rule out functional impairments in nutrient absorption, and that objective measures of intestinal integrity and absorptive capacity may be more informative in evaluating dehydration-induced changes in protein supplementation efficiency.

### 4.3. Mechanisms Underlying the Reduced Amino Acid Response in DEH

The attenuated serum amino acid response observed in DEH likely results from a convergence of multiple gastrointestinal, hemodynamic, and metabolic mechanisms, which can be conceptually organized into four interrelated pathways.

First, exercise-induced splanchnic hypoperfusion is a well-documented phenomenon during endurance exercise, leading to enterocyte injury, increased intestinal permeability, and impaired nutrient absorption [50]. van Wijck et al. demonstrated that greater exercise-related enterocyte injury was accompanied by an approximately 22% lower peak appearance rate of dietary protein-derived phenylalanine during recovery [51]. Because the exercise protocol was identical across conditions in the present study, this exercise-related gut stress was common to all trials; however, the additional fluid deficit in DEH may have exacerbated this effect. Costa et al. directly compared programmed drinking with complete water restriction during 2 h of running and reported greater I-FABP responses and carbohydrate malabsorption when BM loss increased from approximately 0.6% to 3.1% [49]. This suggests that the degree of dehydration compounds the exercise-induced intestinal insult in a dose-dependent manner.

Second, gastric emptying may be delayed under hypohydrated conditions, reducing the rate at which the ingested whey protein beverage is delivered to the small intestine for absorption. Van Nieuwenhoven et al. reported slower liquid gastric emptying after approximately 3% dehydration [11], and Neufer et al. and Rehrer et al. observed impaired gastric emptying when larger fluid deficits were combined with running or heat strain [52,53]. In the present DEH trial, the fluid deficit developed during running, and no fluid was consumed prior to the post-exercise protein beverage, meaning that gastric emptying may have been compromised at the time of protein ingestion. By contrast, Ryan et al. reported no reduction in gastric emptying with 2.7% hypohydration during moderate cycling [54]; however, hypohydration in that study had been established 12–16 h before testing, and participants repeatedly consumed fluid before and during exercise (total intake ~1.83 L), which likely maintained gastric volume. The distinct temporal pattern of fluid deficit development in the present study (acute, exercise-induced, with no pre-ingestion fluid) may therefore explain why gastric emptying effects were more pronounced.

Third, intestinal absorption itself may be directly impaired by hypohydration [49,51]. The absorption of amino acids across the intestinal epithelium relies on sodium-dependent co-transporters (e.g., SGLT1 for glucose, and peptide transporter 1, PEPT1, for di- and tri-peptides) [55,56]. These transport systems are driven by electrochemical gradients maintained by Na^+^/K^+^-ATPase activity at the basolateral membrane, which is energetically demanding and susceptible to alterations in cellular hydration status. Hypohydration may reduce intestinal blood flow and oxygen delivery [57,58], impairing ATP-dependent transport processes and thereby reducing the efficiency of amino acid uptake. This mechanism would manifest as a reduced rate and total amount of amino acids entering the portal circulation, consistent with the attenuated concentration–time responses and lower %iAUC observed in DEH.

Fourth, splanchnic first-pass extraction of amino acids may increase under dehydrated conditions [59]. The liver and splanchnic tissues extract a substantial fraction of dietary amino acids from the portal blood before they reach the systemic circulation. Altered splanchnic perfusion, hormonal milieu (e.g., elevated vasopressin, angiotensin II), and metabolic demands during dehydration could enhance this first-pass extraction, further reducing the peripheral appearance of ingested amino acids [60,61,62]. Although the present study did not measure portal or hepatic amino acid fluxes, this mechanism warrants investigation in future research employing tracer methodologies.

The absence of significant differences in amino acid responses between EUH_50% and EUH is an intriguing finding that merits further consideration. Despite post-exercise BM losses of 1.18% versus 0.27%, representing a nearly four-fold difference in fluid deficit, increasing fluid replacement from 50% to 100% did not yield additional improvements in amino acid availability. This pattern suggests the existence of a threshold or “sufficient hydration” zone, wherein avoiding severe dehydration (i.e., keeping BM loss below approximately 1.2%) is sufficient to preserve protein absorption and amino acid kinetics, whereas achieving “full” hydration offers little added benefit. This is consistent with the observations of Costa et al., who found that complete water restriction resulted in greater intestinal injury than programmed drinking, but that further increases in fluid intake beyond programmed volumes did not proportionally reduce injury markers [49]. From a practical standpoint, this implies that athletes who replace 50% of exercise-induced fluid losses—a level many athletes can achieve without excessive drinking—may already preserve most of the post-exercise whey protein supplementation benefit. However, the present data do not identify the precise inflection point at which dehydration begins to impair amino acid responses; this threshold requires further investigation.

### 4.4. Practical Implications and Limitations

This is the first study to specifically quantify the impact of graded exercise-induced dehydration on serum amino acid responses to whey protein supplementation. The present findings have clear practical implications. Athletes should aim to prevent dehydration exceeding approximately 1.2% BM loss during exercise to preserve the efficacy of subsequent protein intake. For those who do exceed this threshold, increasing the fluid volume of the protein supplement or delaying ingestion until partial rehydration has been achieved may be pragmatic strategies. Importantly, replacing approximately half of fluid losses appears sufficient to preserve the acute amino acid response, alleviating the need for athletes to achieve “perfect” hydration during training to gain the benefits of post-exercise protein supplementation while avoiding the risks of overdrinking [25]. Additionally, the absence of GI discomfort should not be interpreted as a guarantee of optimal nutrient absorption under dehydrated conditions.

Several limitations of the present study should be acknowledged. The sample consisted exclusively of healthy young males, limiting generalizability to females, older adults, or clinical populations. The study did not include direct measurements of gastric emptying, intestinal permeability, or splanchnic blood flow, which would have provided mechanistic confirmation of the proposed pathways. The post-exercise observation period was limited to 120 min, which may not capture full amino acid kinetic profiles, and muscle protein synthesis was not directly assessed. The DEH trial was not randomized, introducing potential order effects, although the 7-day washout and subsequent randomization of the other trials mitigate this concern.

Future studies should address these limitations by incorporating direct measures of gastrointestinal transit and absorption, extending observation periods, including diverse populations, and evaluating different protein doses and sources. Isotopic tracer methodologies would also help distinguish between absorption and utilization effects, while repeated-day dehydration protocols would determine whether these acute effects translate into meaningful differences in chronic training adaptations.

## 5. Conclusions

In conclusion, exercise-induced hypohydration of approximately 2.2% of BM attenuates the early circulating amino acid response to post-exercise whey protein ingestion in healthy young men. These findings indicate that hydration status can influence the effectiveness of post-exercise protein supplementation. Fluid intake during exercise should therefore be maintained to avoid substantial body-water deficits before post-exercise protein intake. This may help preserve amino acid availability and support early post-exercise recovery.

## Figures and Tables

**Table 1:** Hydration, cardiovascular, perceptual, and sweat responses across trials.

Endpoint	DEH	EUH_50%	EUH	ANOVA
Absolute BM loss (kg)	1.72 ± 0.20	0.93 ± 0.26	0.22 ± 0.28	F = 167.86; *p* < 0.001
Relative BM loss (%)	2.20 ± 0.19	1.18 ± 0.30	0.27 ± 0.35	F = 149.28; *p* < 0.001
Mean exercise heart rate (beats/min)	172.3 ± 5.5	168.6 ± 4.1	168.7 ± 4.1	F = 2.71; *p* = 0.080
Mean exercise thirst	5.5 ± 1.3	3.9 ± 1.5	3.5 ± 1.7	F = 6.66; *p* = 0.003
Local sweat rate (g/m^2^/min)	6.55 ± 2.14	6.33 ± 1.49	5.80 ± 1.30	F = 2.13; *p* = 0.141
Sweat sodium (mmol/L)	73.2 ± 24.7	68.4 ± 22.1	67.3 ± 22.1	F = 1.57; *p* = 0.229
Sweat potassium (mmol/L)	5.2 ± 0.9	5.0 ± 1.0	4.7 ± 0.7	F = 2.10; *p* = 0.144
Sweat chloride (mmol/L)	53.5 ± 20.8	50.6 ± 19.8	48.5 ± 20.2	F = 1.16; *p* = 0.329

Values are mean ± SD. For absolute and relative BM loss, all pairwise comparisons were significant (all *p* < 0.001). BM, body mass; DEH, dehydration trial; EUH_50%, 50% fluid replacement trial; EUH, 100% fluid replacement trial; ANOVA, analysis of variance.

**Table 2:** Combined raw scores for each GI symptom category and individual GI symptoms after whey protein ingestion.

Section	Symptom	DEH	EUH_50%	EUH
Upper GI tract (54)		0 (23)	0 (17)	0 (24)
	Reflux	0 (5)	0 (6)	0 (7)
	Heartburn	0 (6)	0 (4)	0 (7)
	Vomiting	0 (3)	0 (2)	0 (2)
	Nausea	0 (4)	0 (4)	0 (4)
	Thick saliva	0 (5)	0 (3)	0 (4)
	Belching	0 (6)	0 (4)	0 (5)
Lower GI tract (36)		0 (8)	0 (8)	0 (6)
	Intestinal cramps	0 (3)	0 (1)	0 (1)
	Abdominal pain	0 (1)	0 (1)	0 (1)
	Bloating/flatulence	0 (5)	0 (5)	0 (4)
	Urge to defecate	0 (3)	0 (7)	0 (3)
Systemic (36)		0 (10)	0 (7)	0 (8)
	Muscle cramping	0 (3)	0 (3)	0 (2)
	Headache	0 (2)	0 (1)	0 (2)
	Urge to urinate	0 (4)	0 (2)	0 (5)
	Dizziness	0 (6)	0 (3)	0 (3)

Values are median (maximum) across the immediate, 60 min, and 120 min post-ingestion assessments.

## Data Availability

Data are contained within the article. The original contributions presented in this study are included in the article. Further inquiries can be directed to the corresponding author.

## Abbreviations

The following abbreviations are used in this manuscript:

%iAUC	percentage incremental area under the curve
AA	amino acid
ANOVA	analysis of variance
AUC	area under the curve
BCAA	branched-chain amino acid
BM	body mass
BM_postexer_	postexercise body mass
BM_pre_	preexercise body mass
DEH	dehydration trial without fluid replacement
EUH	euhydration trial (100% fluid replacement)
EUH_50%	50% fluid-replacement trial
GI	gastrointestinal
HRR	heart rate reserve
I.D.	internal diameter
I-FABP	intestinal fatty acid-binding protein
Iso	isoleucine
LC–MS/MS	liquid chromatography–tandem mass spectrometry
Leu	leucine
ns	not significant
PTFE	polytetrafluoroethylene
RPE	rating of perceived exertion
SD	standard deviation
USG	urine specific gravity
Val	valine
WPI	whey protein isolate
